# MdPHYB2‐MdPIF1 Module Regulates Transcription Factors to Mediate Light‐Dependent ALA‐Induced Apple Anthocyanin Biosynthesis

**DOI:** 10.1111/pce.70702

**Published:** 2026-07-01

**Authors:** Liuzi Zhang, Yuyan An, Jianting Zhang, Liangju Wang

**Affiliations:** ^1^ College of Horticulture Nanjing Agricultural University Nanjing China; ^2^ College of Life Sciences Shaanxi Normal University Xi'an China

**Keywords:** ALA, anthocyanin accumulation, light signalling, MdPHYB2, MdPIF1

## Abstract

Light is a primary environmental signal that induces plant anthocyanin accumulation. The plant growth regulator 5‐aminolevulinic acid (ALA) has also been proven to effectively promote anthocyanin accumulation in apple. However, the underlying regulatory mechanisms remain unknown. In this study, apple phytochrome B2 (MdPHYB2) was identified as the key phytochrome involved in the transcriptional response to ALA under light conditions. Overexpressing (OE)‐*MdPHYB2* promoted anthocyanin accumulation, while RNA interfering (RNAi)‐*MdPHYB2* suppressed it. Library screening and a series of validation tests proved that apple phytochrome‐interacting factor 1 (MdPIF1) is an interactive protein of MdPHYB2, which can interact with each other only under light conditions. OE‐*MdPIF1* inhibited anthocyanin accumulation, while RNAi‐*MdPIF1* promoted it. MdPIF1 can directly bind to E‐box motifs of the promoters of *MdWRKY71*, *MdMADS1*, and *MdNAC33* to repress all gene expression, but not to the promoters of *MdPAL*, *MdCHS*, *MdF3H*, and *MdUFGT*, the structural genes of anthocyanin biosynthesis. Combined with the previous findings, we propose that MdPIF1 is a primary repressor, negatively transcriptionally regulating the expression of *MdWRKY71*, *MdMADS1* (responsible for the non‐MBW route of anthocyanin biosynthesis) and *MdNAC33* (responsible for the MBW route). ALA upregulated *MdPHYB2* but depressed *MdPIF1* expression under light conditions, then cytoplasmic MdPHYB2 was shifted into the nucleus, interacting with MdPIF1 and antagonizing the binding with the promoters of downstream genes, alleviating the gene repression and activating anthocyanin biosynthesis. These findings establish an MdPHYB2‐MdPIF1 module as a central regulatory hub that coordinates light perception and ALA signalling to promote apple anthocyanin biosynthesis.

## Summary statement

In apple, we discover that the MdPHYB2‐MdPIF1 module integrates light and ALA signals to control anthocyanin biosynthesis. Light activates MdPHYB2, which interacts with MdPIF1 in the nucleus to relieve its repression of downstream genes and promote pigmentation.

## 1 Introduction

Light is a critical environmental signal and energy source that drives plant photosynthesis and serves as a multifaceted regulator of diverse plant growth and developmental processes, including seed germination (Franklin and Whitelam 2004; Zeidler 2022), cotyledon expansion (Zhao et al. 2023), de‐etiolation (Iglesias‐Sanchez et al. 2024; Mu et al. 2024), and anthocyanin biosynthesis (Bai et al. 2019; Liu, Shu, et al. 2021). To perceive these signals, plants have evolved many photoreceptors, such as phytochromes (PHYs) for red/far‐red light, UVRESISTANCE LOCUS8 (UVR8) for ultraviolet B (UV‐B) light, cryptochromes (CRYs) and phototropins for blue light (Cheng et al. 2021; Chen et al. 2022; Qu et al. 2024). Among these, PHYs have been most extensively studied (Rockwell et al. 2006; Liu, Weng, et al. 2021).

In higher plants, the diversity of PHY types is underpinned by a small, multigene family. As described by Quail (1998), the PHY family in *Arabidopsis thaliana* comprises five members: AtPHYA‐AtPHYE, where PHYA is light lability, whereas the others from PHYB to PHYE are light‐stable (Jung et al. 2016). PHYs typically exist as dimers and undergo light‐induced transformation between the inactive Pr form, localized in the cytoplasm, and the active Pfr form, when they are translocated into the nucleus (Rockwell et al. 2006). Once in the nucleus, Pfr transduces the light signal and initiates light‐dependent photomorphogenesis (Mu et al. 2024). Accumulating evidence indicates that PHYs fundamentally mediate light‐regulated anthocyanin accumulation. For instance, in potato (Yanovsky et al. 1998) and *A. thaliana* (Li et al. 2014), PHYA functions as a positive regulator of anthocyanin biosynthesis. Likewise, in tomato (*Solanum lycopersicum*), overexpression of *SlPHYB2* led to a significant increase in anthocyanin biosynthesis and potentiated the red‐light response (Husaineid et al. 2007). In woodland strawberry (*Fragaria vesca*), the *FvephyB* mutant exhibits drastically reduced anthocyanin accumulation in petioles and fruits (Gao et al. 2023), reinforcing the conserved role of PHYs across diverse species.

Phytochrome‐interacting factors (PIFs), a subgroup of bHLH transcription factors (TFs), are key partners of PHYs that orchestrate the transition from skotomorphogenesis to photomorphogenesis (Wang et al. 2022). PIFs can integrate diverse environmental signals, positively regulating skotomorphogenesis and negatively regulating photomorphogenesis. Beyond the well‐known roles in shade avoidance and thermomorphogenesis, PIFs have emerged as pivotal regulators of anthocyanin biosynthesis (Wang, Lei, et al. 2025). In *A. thaliana*, PIF4 directly binds to the G‐box motif of the *PAP1* promoter to repress the TF gene expression as well as anthocyanin accumulation (Liu, Wang, et al. 2021), whereas PIF5 primarily represses *DFR* expression to inhibit tissue coloration (Liu et al. 2015). In *Crocus sativus*, CstPIF4 interacts with CstMYB16 to jointly suppress anthocyanin biosynthesis (Hussain et al. 2023). In red pear, light suppresses PyPIF5 to release *PymiR156a*, which then degrades PySPL9 and activates the PyMYB114/MYB10 complex for anthocyanin biosynthesis (Liu, Shu, et al. 2021). Consistent with these, apple MdPIF7 inhibits anthocyanin biosynthesis by interfering with the function of positive regulatory TFs such as MdBBX23 and MdbZIP44/MdERF38 (Liu et al. 2022). However, how MdPHYs and MdPIFs coordinately regulate apple anthocyanin accumulation remains unknown.

5‐Aminolevulinic acid (ALA), the common biosynthetic precursor of tetrapyrroles (including phytochromobilin), is widely used as a plant growth regulator, which can induce anthocyanin accumulation across various fruits (Wang et al. 2023; Wang, Zhang, Zhao, et al. 2025). In apple, ALA upregulates expression of the structural genes (such as *CHS*, *DFR*, and *UFGT*) and TF genes (such as *MYB*, *bHLH*, and *WD40*) during fruit coloration (Xie et al. 2013). Feng et al. (2016) identified MdMADS1 as a positive regulator in ALA‐induced anthocyanin accumulation, while MdMYB9 and MdMYB10 facilitate transport by binding to the *MdMATE8* promoter (Zheng et al. 2021). MdERF78, another ALA‐responsive TF, directly activates *MdF3H* and *MdANS* expression to mediate anthocyanin accumulation (Fang et al. 2022). Similarly, MdMYB110a regulates ALA‐induced anthocyanin biosynthesis through different pathways (Fang et al. 2023). Recently, Zhang, Zhang, et al. (2024) reported that MdNAC33 positively regulates ALA's effect via three mechanisms: (1) direct activation of *MdDFR* and *MdANS*, (2) transcriptional upregulation of *MdbHLH3* to increase *MdDFR* and *MdUFGT* transcription, and (3) protein–protein interaction with MdMYB1 to enhance *MdUFGT* and *MdGSTF12* expression, thus operating through an MYB‐bHLH‐WD40 (MBW) pathway. Furthermore, Zhang et al. (2025) proposed an MBW‐independent pathway in which ALA induces an MdWRKY71‐MdMADS1 module: MdWRKY71 transcriptionally activates *MdMADS1*, and both TFs synergistically upregulate *MdDFR*, *MdANS*, *MdCHS*, and *MdUFGT*. These findings establish two distinct signalling pathways in ALA‐induced apple anthocyanin accumulation, yet the regulatory mechanisms upstream of these TFs (*MdNAC33*, *MdWRKY71* and *MdMADS1*) remain to be elucidated. When Wang et al. (2004) initially reported that ALA induced apple anthocyanin accumulation, they hypothesized that PHYs might be involved, given that ALA is the essential precursor of phytochromobilin. However, the roles of PHYs in woody plants, such as apple, remain largely unexplored (Ma et al. 2019). We speculate that MdPHYs and MdPIFs are involved in the aforementioned regulation mechanism of ALA.

In this study, we identified the *MdPHY* gene family in the apple genome and examined their protein–protein interactions and light‐dependent subcellular translocation, revealing MdPHYB2 as a key positive regulator of ALA‐induced anthocyanin biosynthesis. Using a yeast two‐hybrid (Y2H) library screen, we identified MdPIF1 as a downstream interacting partner of MdPHYB2. This interaction relieves MdPIF1‐mediated repression on downstream TF genes, including *MdWRKY71*, *MdMADS1* and *MdNAC33*, thereby activating the structural and transport genes to promote anthocyanin accumulation. These findings provide essential insights into the molecular mechanism underlying ALA‐regulated fruit coloration and significantly advance our understanding of the roles of MdPHYB2 and MdPIF1 in apple anthocyanin biosynthesis.

## 2 Materials and Methods

### 2.1 Whole‐Genome Identification of the *MdPHY*s in Apple

To identify *MdPHYs*, we downloaded the *A. thaliana PHYs* from the TAIR website (http://www.arabidopsis.org/). Using these sequences as queries, we performed BLASTp against the apple genome (GDDH13 Version 1.1) protein database. The identified homologous genes were confirmed as *MdPHY* family members using the PFAM and SMART software. The Expasy toolkit (https://www.expasy.org/) and the Plant‐mPloc server (http://www.csbio.sjtu.edu.cn/bioinf/plant-multi/) were employed to predict the molecular properties and subcellular localization of MdPHY proteins (Supporting Information S2: Table S1).

### 2.2 Phylogenetic Analysis, Chromosomal Localization and Synteny Analysis of *MdPHYs*

For phylogenetic analysis, we collected PHY protein sequences from multiple species: apple, *A. thaliana*, *Camellia sinensis* (tea), *Nicotiana tabacum* (tobacco), *Oryza sativa* (rice), *Prunus persica* (peach), *S. lycopersicum* (tomato), *Vitis vinifera* (grape), and *Zea mays* (maize), resulting in a dataset of 47 sequences (Supporting Information S2: Table S2). These sequences were aligned using DNAMAN software, and the phylogenetic tree was constructed with MEGA‐X and visualized with iTOL (https://itol.embl.de/). To determine chromosomal locations, we retrieved *MdPHY* gene positions from the apple genome annotation file (Rosaceae Genome Database). The *MdPHY* genes were mapped to chromosomes using TBtools. For synteny analysis, we identified homologous gene pairs among *MdPHY* genes by performing an all‐against‐all BLASTp search. We then used TBtools to generate a Circos plot, illustrating the distribution of syntenic gene pairs (Chen et al. 2023).

### 2.3 Conserved Domains, Gene Structure and *cis‐*Acting Element Analysis of *MdPHYs*

The conserved domains of MdPHYs were identified using the NCBI CDD tool (https://www. ncbi.nlm.nih.gov/cdd/). We retrieved the gene structure information of the seven *MdPHYs* from the Apple Genome Database (GDR) and visualized them using TBtools software. Then, the putative promoter sequence of *MdPHYs* was extracted (about 2000 bp). The PlantCARE (http://bioinformatics.psb.ugent.be/webtools/plantcare/html/) was used to predict *cis*‐acting regulatory elements within these promoter regions (Supporting Information S2: Table S3). Visualization mapping was performed with TBtools software.

### 2.4 Plant Materials and Treatments

For ALA treatment and transient transformation experiments, mature bagged fruits of apple (*M*. × *domestica* cv. Gala, Huashuo, or Fuji) were used. ALA solution in a dosage of 200 mg L^−1^ was used to soak apple fruits for 5 s, a concentration based on previous studies (Xie et al. 2013; Zhang et al. 2025), as effective for penetrating the dense fruit tissues and inducing a consistent anthocyanin response. Then, the fruits were illuminated in a growth chamber with a continuous light intensity of 200 μmol m^−2^ s^−1^ at 17°C, which were taken at 24, 48, 72 h, respectively, for photograph, and the peels were collected and frozen at −80°C for the following measurements.

Apple calli (*M*. × *domestica* cv. Orin) were cultured as previously described by Fang et al. (2022, 2023). For ALA treatment, the culture medium was added with 250 μg L^−1^ ALA (filter sterilization), a concentration optimized for the loose, readily absorbing callus tissue (Zhang, Zhang, et al. 2024). After 2 weeks of culture, the anthocyanin content and gene expression were analyzed.

The detached leaves were obtained from the tube plantlets of apple (*M*. × *domestica* cv. GL‐3), which were cultured on MS solid medium as described previously (Zhang, Zhang, et al. 2024). For ALA treatment, leaves were cultured on MS medium containing 25 μg L^−1^ ALA (filter sterilization), a concentration identified as optimal for the highly sensitive leaf tissue (Zhang et al. 2025). After 3 days of continuous light culture (200 μmol m^−2^ s^−1^) at 24°C, the anthocyanin content and gene expression were analyzed.

### 2.5 Construction of Expression Vectors and Genetic Transformation

To construct overexpression vectors for the target genes (*MdPHYB2* and *MdPIF1*), we cloned their coding sequences (CDSs) and inserted them into the pCAMBIA1300 vector. RNA interference (RNAi) vectors were constructed by inserting specific fragments of *MdPHYB2* and *MdPIF1* into the pHELLSGATE2 (pHG2) plasmid using the Gateway BP cloning system (Thermo Fisher Scientific, USA). The fragment for *MdPHYB2* was specifically designed from a region with low homology to *MdPHYB1* to avoid off‐target silencing. The sequences of all primers used in this study for vector construction are listed in Supporting Information S2: Table S4.

Apple calli were transformed according to the previous methods (Fang et al. 2022). Briefly, calli were co‐cultivated with *Agrobacterium* LBA4404 harbouring the constructed vectors, then screened and cultured on MS medium with appropriate antibiotics (Zhang, Zhang, et al. 2024). Successful transformants were confirmed by PCR and RT‐qPCR, with at least three biological replicates per genotype.

Apple fruit injection was performed according to the method previously reported (Zhang, Zhang, et al. 2024) with the empty vector (EV) as the control and treated with 200 mg L^−1^ ALA or not. Each treatment had at least three biological replicates, and the fruits were illuminated in the growth chamber with continuous light intensity of 200 μmol m^−2^ s^−1^ at 17°C. Three days later, the gene expression and anthocyanin content were analyzed.

For the transient transformation of ‘GL‐3’ apple leaves, the detached leaves were immersed in *Agrobacterium* EHA105 suspension carrying the recombinant vectors. They were then subjected to vacuum infiltration for 5 min, repeated twice, and kept in the dark for 24 h. After 3 days of illumination, the leaves were collected and stored for subsequent analysis (Zhang, Zhang, et al. 2024).

### 2.6 RNA Extraction and RT‐qPCR Analysis

Total RNA was extracted from different apple materials using an RNA extraction kit (Vazyme, China) following the manufacturer's instructions. The cDNA was synthesized using a reverse transcription kit (Transgen, China). RT‐qPCR was performed using the 2× SYBR Green Pro Taq HS Premix* (Accurate Biotech. Co. Ltd., Changsha, China). The relative gene expression levels were calculated by the 2^−ΔΔCT^ method (Livak and Schmittgen 2001) with the primers in Supporting Information S2: Table S4.

### 2.7 Determination of the Anthocyanin Content

For anthocyanin measurement, samples were ground into a fine powder in liquid nitrogen and homogenized with 1 mL of 1% (v/v) HCl‐methanol solution. The homogenate was incubated at 4°C in the dark for 24 h. Anthocyanin absorbance at 530, 620 and 650 nm was determined, and the anthocyanin content was calculated according to the previous report (Zhang et al. 2025).

### 2.8 Subcellular Localization Analysis

The CDSs of *MdPHYs* and *MdPIF1* were cloned into the pCAMBIA1300‐GFP vector. The recombinant vectors were transformed into tobacco leaves using *Agrobacterium* GV3101 (Zhuge et al. 2023). Fluorescence signal was observed 2 days later using a confocal microscope (LSM 780, Zeiss). For dark‐condition localization, fluorescence was detected 2 days after transient injection in the dark. For light‐treated localization, tobacco plants were kept in the dark for 2 days, then exposed to light (100 μmol m^−2^ s^−1^, 24°C) for 6 h before fluorescence detection.

### 2.9 Y2H

We constructed the bait and prey vectors by cloning the *MdPHYs* CDS into pGBKT7 and the CDSs of both *MdPHYs* and *MdPIFs* into pGADT7, respectively. The bait and prey vectors were co‐transformed into Y2HGold cells and cultured on SD/−T/−L (−Tryptophan/−Leucine) medium for 3 days. Subsequently, the positive clones were dotted on the quadruple dropout selective medium SD/−T/−L/−H/−A (−Tryptophan/−Leucine/−Histidine/−Adenine) to observe yeast growth and protein–protein interactions.

### 2.10 Bimolecular Fluorescence Complementation Assays (BiFC)

For BiFC, the CDS of *MdPHYB2* was inserted into the pSPYNE vector to generate nYFP‐MdPHYB2. The CDSs of *MdPIFs and MdPHYB2* were cloned into the pSPYCE vector to generate cYFP‐MdPIFs and cYFP‐MdPHYB2, respectively. These vectors were transformed into GV3101 and then injected into tobacco. After 2 days, confocal microscopy was used to detect the YFP signal.

### 2.11 Luciferase Complementation Imaging (LCI) Assay

The *MdPHYB2* CDS without stop codon was inserted into the pCAMBIA1300‐nLUC vector to construct *35S::MdPHYB2‐nLUC*. The CDSs of *MdPIF1* and *MdPHYB2* were inserted into pCAMBIA1300‐cLUC to construct *35S::cLUC‐MdPIF1* and *35S::cLUC‐MdPHYB2*, respectively. These vectors were transformed into GV3101 and then injected into tobacco. After 2 days, the LUC fluorescence intensity was detected and quantified with a PIXIS 1024B imager (USA).

### 2.12 Yeast One‑Hybrid (Y1H)

Approximately 2000 bp promoter fragments of the aforementioned genes were cloned into the pAbAi vector. AD‐*MdPIF1* vector was transformed into yeast competent cells containing these promoters. The SD/−Leu medium, supplemented with Aureobasidin A (AbA), was used as the selection medium.

### 2.13 Electrophoretic Mobility Shift Assay (EMSA)

The *MdPIF1* CDS was inserted into the His‐tagged pET‐32 vector to obtain a recombinant plasmid, and then transformed into *Escherichia coli* BL21 (DE3) cells for fusion protein production. The fusion protein was purified using a protein purification kit (CWbio, China), and EMSA was performed using an EMSA kit (Thermo Fisher Scientific, USA), as referred in the previous report (Zhang et al. 2025).

### 2.14 GUS Staining Determination

Histochemical staining was used to detect promoter activity. Promoter fragments, about 2000 bp of the aforementioned genes, were individually fused into the pBI121‐GUS vector. The CDSs of *MdPHYB2* and *MdPIF1* were cloned into the pCAMBIA1300 as effector vectors. These vectors were transformed into GV3101 and injected into tobacco. After 2 days, GUS staining was performed as previously described in Fang et al. (2023).

### 2.15 Luciferase Reporter Gene Detection

The CDSs of *MdPHYB2* and *MdPIF1* were cloned into the pCAMBIA1300 to generate effector constructs. The promoters of TF and the structural genes were individually cloned into pGreenII0800‐LUC as reporters. Vectors were transformed into GV3101 (pSoup) and injected into tobacco. After 2 days, we detected and quantified the LUC fluorescence intensity with a PIXIS 1024B imager (USA).

### 2.16 Statistical Analysis

Data are presented as means ± standard errors (SEs). For comparisons between two groups, Student's *t*‐test was employed. For comparisons among three or more groups, statistical significance was assessed using one‐way or two‐way analysis of variance (ANOVA), followed by Tukey's honestly significant difference test for mean separation.

## 3 Results

### 3.1 Genome‐Wide Identification of the *MdPHY* Genes in Apple

To identify PHY family members in the apple genome, a BLASTp analysis was conducted using the protein sequences of *Arabidopsis* PHYs (PHYA to PHYE) as queries. Seven putative MdPHY candidates were identified across seven chromosomes (Supporting Information S1: Figure S1A, Supporting Information S2: Table S1). The MdPHY proteins ranged in molecular weights from 124.06 to 129.07 kDa with isoelectric points of 5.50–5.84. The instability indexes ranged from 41.63 to 53.80, suggesting that they are unstable proteins. All MdPHY proteins showed negative grand average of hydropathicity (GRAVY) scores, consistent with hydrophilic properties. The subcellular localization prediction showed that all MdPHYs were dual‐targeted to the cytoplasm and nucleus (Supporting Information S2: Table S1).

Phylogenetic analysis of nine plant species showed that apple PHY members clustered into four distinct subgroups: PHYA, PHYB, PHYC and PHYE, lacking PHYD, which was present in *Arabidopsis* and tobacco but absent from apple, peach and grape (Supporting Information S1: Figure S1B). Among MdPHYs, MdPHYA1 and MdPHYA2 belonged to subgroup A, MdPHYB1 and MdPHYB2 to subgroup B, MdPHYC to subgroup C, and MdPHYE1 and MdPHYE2 to subgroup E (Supporting Information S1: Figure S1C). Notably, MdPHYs exhibited a close phylogenetic relationship with peach PpPHYs, consistent with their shared membership in the Rosaceae family.

To validate the phylogenetic classification of MdPHYs, we analyzed their conserved domains and gene structures. Utilizing the NCBI CDD online tool, we found that all MdPHYs contained a Period/Arnt/Single‐Minded_2 (PAS_2) domain, a cGMP phosphodiesterase/adenylyl cyclase/FhlA (GAF) domain, which binds phytochromobilin, and a phytochrome‐specific (PHY) domain at the N‐terminus (Supporting Information S1: Figure S1C). At the C‐terminus, two tandem PAS domains and a histidine kinase‐related domain (HKRD) were present, which further comprises a Histidine Kinase A/phosphoacceptor (HisKA) domain and a Histidine kinase‐like ATPases (HATPase_c) domain. Next, we analyzed the exon‐intron structure of *MdPHY* genes and found that exon numbers ranged from 3 to 5 and intron numbers from 4 to 6, with consistent counts within each subgroup. In addition, we identified three pairs of homologous genes within the *MdPHY* family, with only *MdPHYC* located on chromosome 9 of the apple genome (Supporting Information S1: Figure S1D).

To investigate potential functions of MdPHYs, we examined their promoter regions (2000 bp upstream) for *cis*‐acting elements (Supporting Information S2: Table S3). All *MdPHY* promoters harboured multiple light‐responsive elements (Supporting Information S1: Figure S1E,F), with *MdPHYE2* possessing the highest number (20) and *MdPHYB2* the lowest (7). Some promoters also contained *cis*‐acting elements responsive to other environmental stimuli (low temperature, drought) and phytohormones (ABA, auxin, gibberellin, methyl‐jasmonic acid [MeJA], salicylic acid). Notably, 85.71% of *MdPHY* genes contained ABA‐responsive elements, implying their potential involvement in ABA‐mediated responses.

### 3.2 MdPHYs Form Specific Dimers and Undergo Light‐Induced Nuclear Import

Phytochromes typically function as homo or heterodimers (Clack et al. 2009). We cloned the seven *MdPHYs* into both activation and binding domain vectors for Y2H assays to test potential interactions. Self‐activation assays showed that none of the MdPHYs have autoactivation (Figure 1A). The pairwise Y2H screening showed that MdPHYA1 and MdPHYC can form only homodimers, while the other members can form multiple interactions (Figure 1B). Notably, MdPHYB2 can interact with MdPHYA2, MdPHYB1, and MdPHYE1 as well as itself under dark conditions. Furthermore, we performed Y2H, LCI and BiFC to verify MdPHYB2 self‐interaction under light conditions (Supporting Information S1: Figure S2). The results showed that MdPHYB2 can self‐interact in yeast under light. This suggests that MdPHYB2 homodimer formation is not light‐dependent. The self‐interaction of MdPHYB2 was further verified in tobacco leaves by LCI and BiFC. Notably, in BiFC, tobacco leaves exhibited a yellow spot formed by MdPHYB2 self‐interaction upon illumination, which suggests that the homodimer may reside within the cell nucleus.

**Figure 1:**
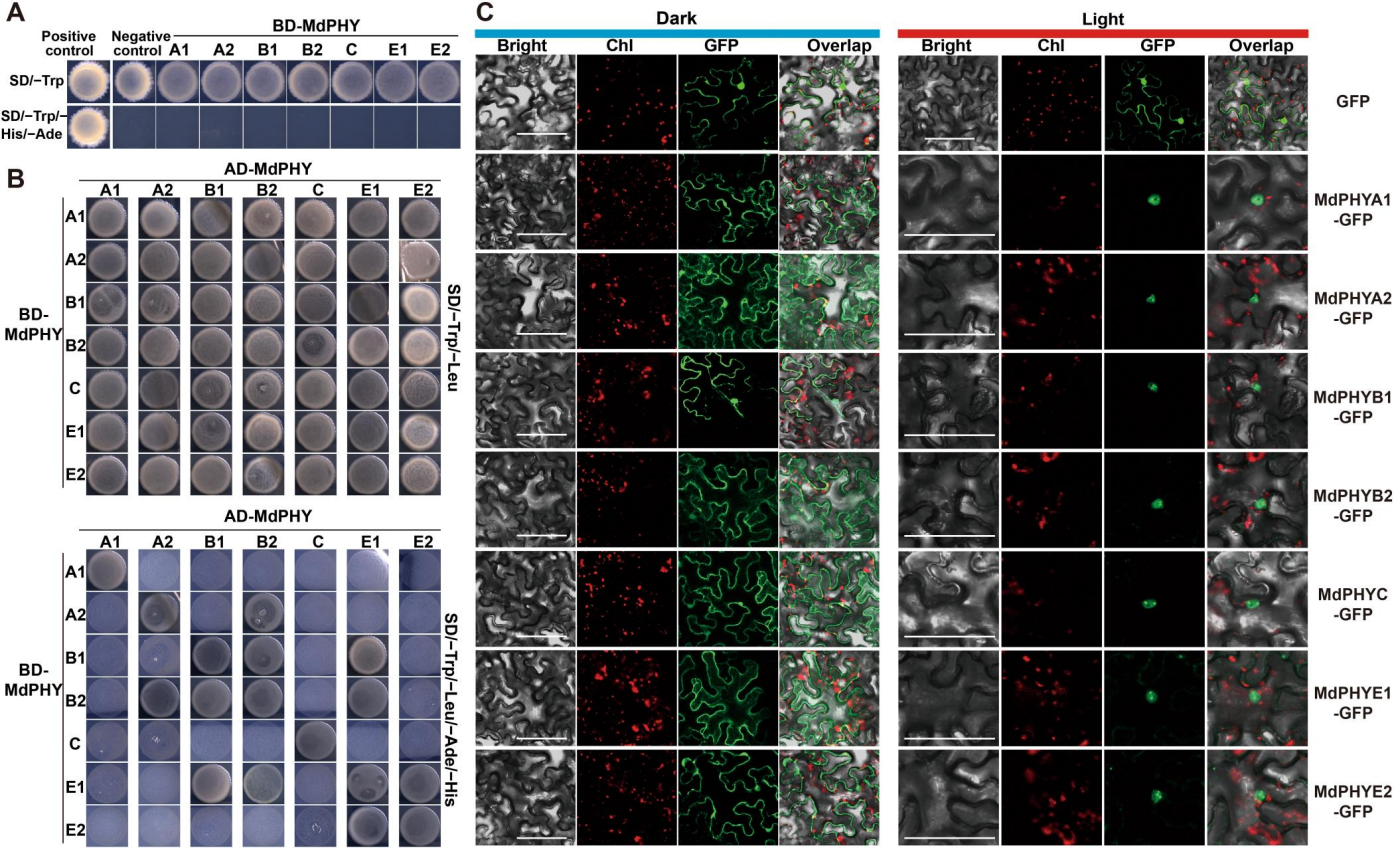
MdPHY member interactions and subcellular localization under dark and light conditions. (A) Self‐activation assay of MdPHY members. The seven MdPHYs were transformed into the yeast strain Y2HGold using the pGBKT7‐MdPHYs (BD‐MdPHYs) vector. Normal growth was observed on SD/−Trp but not on SD/−Trp/−His/−Ade selective media for 3 days. (B) Y2H interaction analysis. Full‐length *MdPHY* sequences were constructed into BD and AD vectors and co‐transformed into Y2HGold yeast. Interactions were assessed on SD/−Trp/−Leu and SD/−Trp/−Leu/−Ade/−His selective media for 3 days in the dark. Representative yeast colonies are presented here. (C) Subcellular localization of MdPHY members in tobacco leaf epidermal cells under dark and light conditions. Scale bar = 20 µm.

Subsequently, to elucidate the subcellular localization of MdPHYs, we expressed MdPHY‐GFP proteins in tobacco leaves. Notably, their localization pattern was light‐responsive: cytoplasmic in darkness and nuclear upon light exposure (Figure 1C). These results demonstrate that MdPHY proteins undergo light‐induced nuclear translocation to transmit light signals and regulate light‐related biological processes.

### 3.3 ALA Promotes *MdPHYB2* Expression and Anthocyanin Accumulation

ALA, a biosynthetic precursor of phytochromobilin, promotes apple coloration (Wang et al. 2004). To assess the role of MdPHYs in this process, we treated detached apple fruits with exogenous ALA and monitored *MdPHYs* expression during peel coloration. Results showed that ALA treatment significantly enhanced anthocyanin accumulation in the peels after 48 h of illumination (Figure 2A,B), but failed to promote coloration in darkness (Supporting Information S1: Figure S3), indicating that light is required for this process. Expression analysis showed that, among the seven *MdPHYs*, only *MdPHYB2* was consistently up‐regulated at all time points, while the other *MdPHYs* had no stable positive responses (Figure 2C, Supporting Information S1: Figure S4). These suggest that MdPHYB2 likely contributes to ALA‐induced anthocyanin synthesis. Subsequently, we conducted an analysis of sequence similarity for the amino acid sequences of PHYB across the nine species. The results revealed that MdPHYB2 shares the highest similarity with PpPHYB from peach and PcPHYB from pear (Figure 2D), suggesting they may possess similar functions.

**Figure 2:**
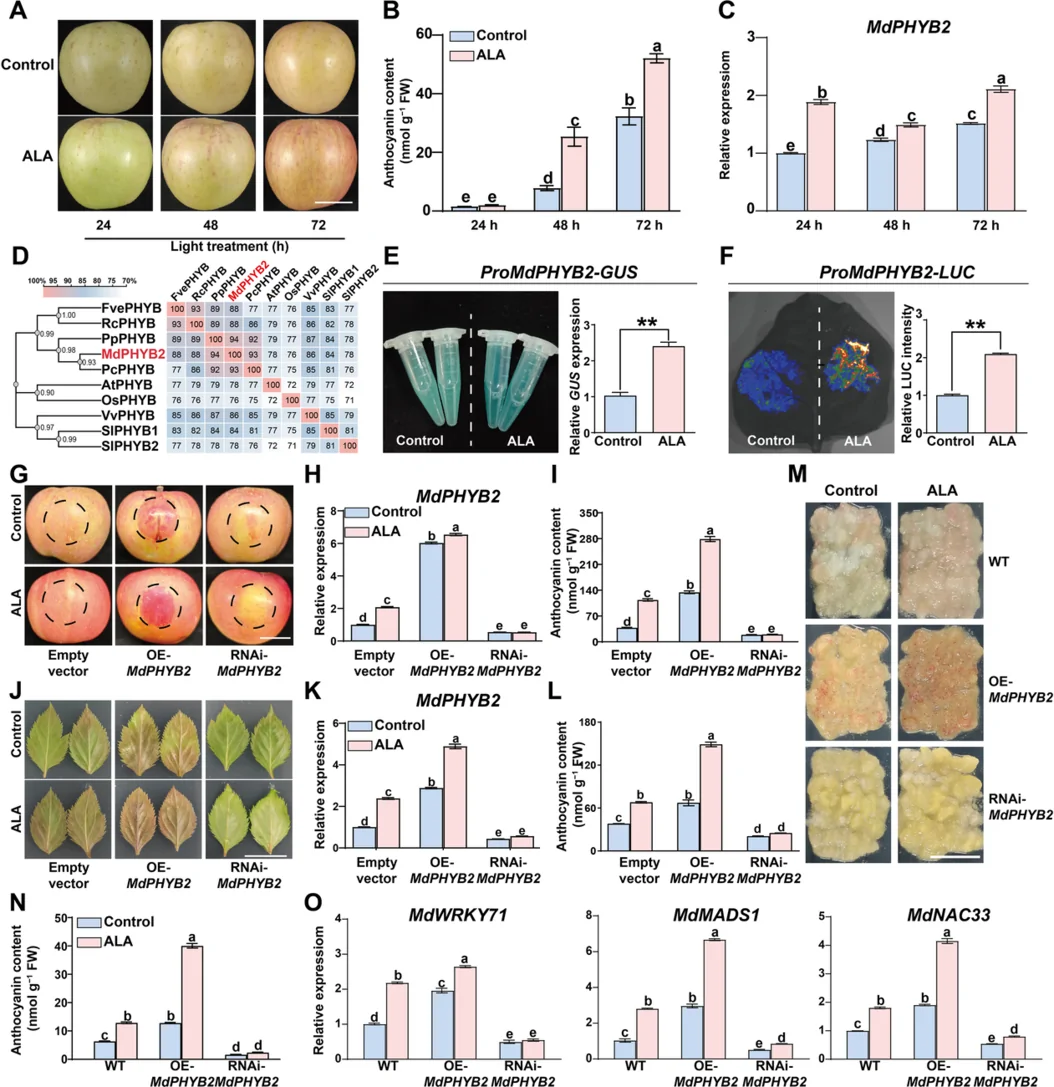
MdPHYB2 contributes to ALA‐induced anthocyanin accumulation in apple. (A) ALA promotes apple coloration under light. Bagged ‘Gala’ apples were debagged and treated with 200 mg L^−1^ ALA at 17°C for 72 h illumination. Scale bar = 4 cm. (B) Anthocyanin content in apple peel of (A). (C) The relative expression of *MdPHYB2* during ALA‐inducing apple coloration under light. (D) Phylogenetic analysis and sequence similarity of MdPHYB2. A phylogenetic tree was constructed based on the amino acid sequences of MdPHYB2 and PHYB orthologs from eight other plant species. The species included are: *Fragaria vesca*, *Rosa chinensis*, *Prunus persica*, *M. × domestica*, *Pyrus communis*, *Arabidopsis thaliana*, *Oryza sativa*, *Vitis vinifera*, and *Solanum lycopersicum*. Heatmap showing the percentage of pairwise amino acid sequence identity among the PHYB proteins. The similarity percentage is indicated both numerically and by the colour gradient in each cell. (E) Effect of ALA on *MdPHYB2* promoter activity by GUS assay in ‘Orin’ calli. The ‘Orin’ calli transformed with *ProMdPHYB2‐GUS* were treated with 250 μg L^−1^ ALA or not (control). *ProMdPHYB2‐GUS* activity and *GUS* expression were shown. (F) Effect of ALA on *MdPHYB2* promoter activity by LUC assay in tobacco leaves, where 0.5 mg L^−1^ ALA was sprayed on the leaves. All experiments were replicated three times. (G) Transient transformation of apple peel. Debagged ‘Fuji’ apple fruits were injected with a carrier, treated with or without 200 mg L^−1^ ALA and then incubated under constant light (200 μmol m^−2^ s^−1^) at 17°C. OE‐*MdPHYB2*, Overexpressing *MdPHYB2*; RNAi‐*MdPHYB2*, Interfering expression of *MdPHYB2*. Fruits were light‐exposed for 5 days to ensure coloration. The dashed circles point to the injection sites. Scale bar = 4 cm. (H) *MdPHYB2* relative expression in the injection sites of (G). (I) Anthocyanin content in the injection sites of (G). (J) Phenotype of OE‐*MdPHYB2* and RNAi‐*MdPHYB2* transiently transformed apple leaves treated with or without 25 μg L^−1^ ALA for 3 days. Scale bar = 1 cm. (K) *MdPHYB2* relative expression in (J). (L) Anthocyanin content in (J). (M) Phenotype of OE‐*MdPHYB2* and RNAi‐*MdPHYB2* stable transgenic ‘Orin’ calli treated with or without 250 μg L^−1^ ALA for 2 weeks. Scale bar = 1 cm. (N) Anthocyanin content in (M). (O) The relative expression of *MdWRKY71*, *MdMADS1*, and *MdNAC33* in (M). All experiments were replicated three times. Values are means ± standard errors (SEs, *n* = 3). The different lowercases in a panel represent significant differences (B, C, H, I, K, L, N and O; two‐way ANOVA followed by Tukey's test, *p* < 0.05). Asterisks indicate significant differences (E and F; Student's *t*‐test, **p < 0.01). [Color figure can be viewed at wileyonlinelibrary.com]

To test whether ALA activates the promoter of *MdPHYB2*, we cloned a 2000 bp promoter fragment to create *ProMdPHYB2‐GUS* and *ProMdPHYB2*‐*LUC* constructs for transient transformation into apple calli and tobacco leaves, respectively. ALA treatment increased GUS staining and gene expression in *ProMdPHYB2‐GUS* apple calli (Figure 2E) and enhanced LUC activity in *ProMdPHYB2‐LUC* tobacco leaves (Figure 2F). Together, these results demonstrate that ALA activates *MdPHYB2* promoter activity and gene expression, linking MdPHYB2 to ALA‐promoted anthocyanin accumulation.

### 3.4 MdPHYB2 Is Required for ALA‐Promoted Anthocyanin Biosynthesis

To verify the function of MdPHYB2 in ALA‐promoted anthocyanin biosynthesis in apple, the *MdPHYB2* overexpression (OE) or RNAi vectors were transformed into three different apple materials, which were then subjected to ALA treatment.

In the transient transformation with detached apple fruits, OE‐*MdPHYB2* caused the infiltration sites to appear redder, with a 2.5‐fold higher anthocyanin content than the EV, while RNAi‐*MdPHYB2* fruits showed a 49.46% reduction (Figure 2G–I). ALA treatment elicited a more pronounced anthocyanin accumulation in the OE‐*MdPHYB2* but had no effect in the RNAi‐*MdPHYB2*. Moreover, similar results were obtained in the transient transformation of ‘GL‐3’ leaves (Figure 2J–L). These results demonstrate that MdPHYB2 is essential for ALA to promote anthocyanin accumulation in apple.

To further substantiate the role of MdPHYB2, we transformed ‘Orin’ calli with OE‐*MdPHYB2* and RNAi‐*MdPHYB2* vectors to generate stable transgenic apple callus lines. After verification via western blot, PCR and RT‐qPCR (Supporting Information S1: Figure S5), the positive lines were subjected to ALA treatment. Additionally, since MdPHYB2 and MdPHYB1 share high sequence similarity, we detected *MdPHYB1* expression in the RNAi‐*MdPHYB2* lines. The results showed that the expression of *MdPHYB1* was not affected in the RNAi‐*MdPHYB2* lines (Supporting Information S1: Figure S6). This confirms that the RNA‐interfering fragment is specific to *MdPHBY2*, not to *MdPHBY1* expression. In the OE‐*MdPHYB2* lines, the anthocyanin accumulation was substantially promoted, particularly in response to ALA, whereas RNAi‐*MdPHYB2* abolished the promotive effect of ALA (Figure 2M,N). Furthermore, we evaluated the expression of key TF genes known to be associated with ALA‐induced anthocyanin accumulation (Zhang, Zhang, et al. 2024; Zhang et al. 2025) in the transgenic calli. The results showed that *MdWRKY71*, *MdMADS1*, and *MdNAC33* expression was significantly upregulated in OE‐*MdPHYB2* lines, but downregulated in the RNAi‐*MdPHYB2* lines (Figure 2O). ALA promoted the gene expression in OE‐*MdPHYB2* but had minimal effect in RNAi‐*MdPHYB2* lines. These results confirm that MdPHYB2 is required for ALA‐induced anthocyanin biosynthesis in apple.

### 3.5 MdPHYB2 Interacts With MdPIF1 in a Light‐Dependent Manner

To elucidate the molecular mechanism by which MdPHYB2 promotes anthocyanin accumulation in apple, we screened a Y2H library under light to identify MdPHYB2‐interacting proteins. Through the screen, we obtained more than 60 proteins (Supporting Information S2: Table S5), among which a gene (KU255859.1/MDP0000289642) designated as apple *phytochrome‐interacting factor1* (*MdPIF1*) was noticed. Since PHYs are reported to transduce signals through PIF interactions (Cheng et al. 2021), we paid attention to this protein. Firstly, Y2H assays showed that co‐transformation of pGADT7(AD)‐MdPIF1 and pGBKT7(BD)‐MdPHYB2 into the Y2HGold strain enabled yeast growth on SD/ − T/ − L/ − H/ − A medium with X‐α‐gal, turning colonies blue only under light conditions (Figure 3A). These demonstrate that an MdPHYB2‐MdPIF1 interaction in yeast is light‐dependent.

**Figure 3:**
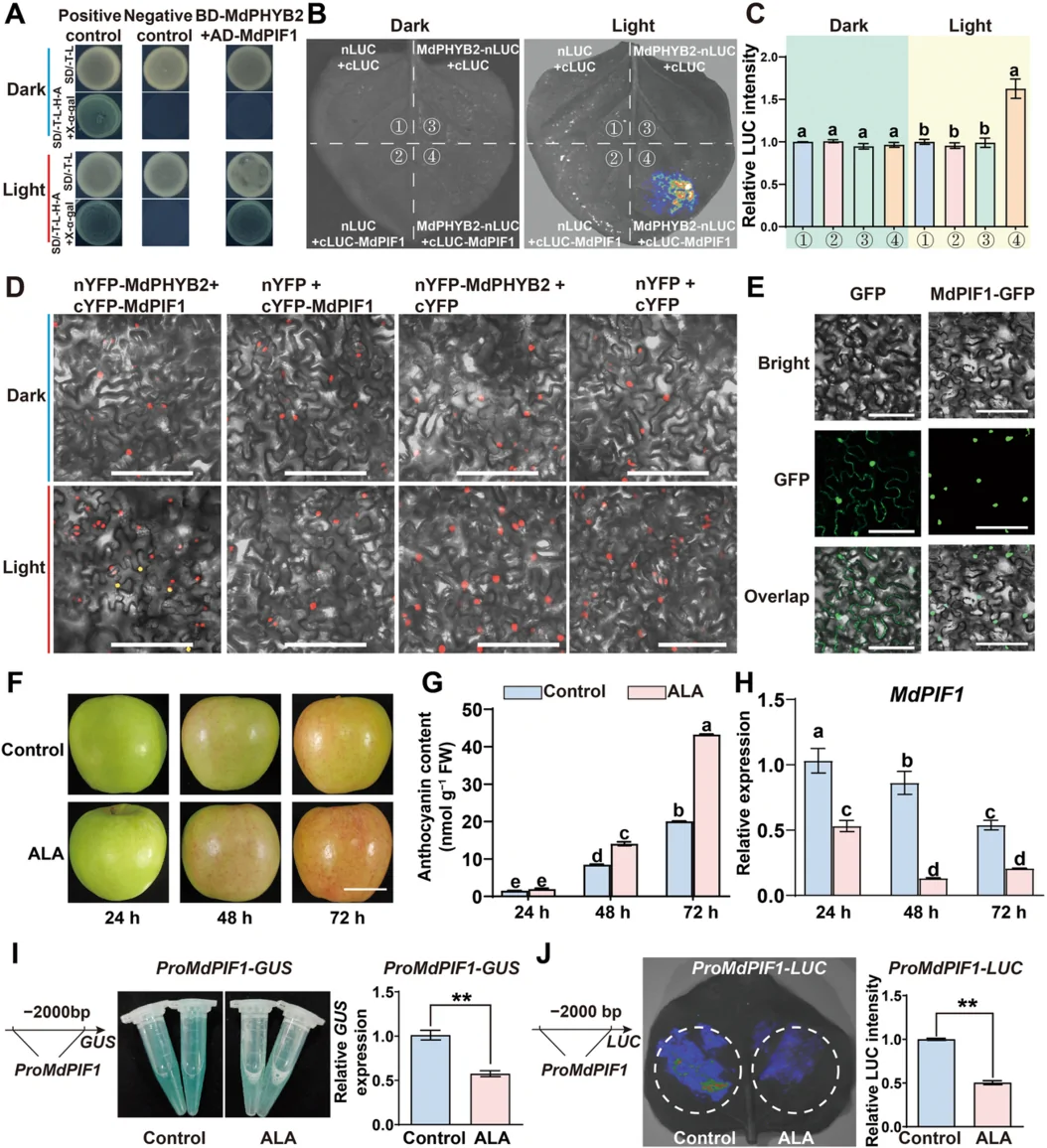
Interaction of MdPHYB2 with MdPIF1 and verification of MdPIF1 function. (A) Interaction between MdPHYB2 and MdPIF1 under dark and light conditions by Y2H. The yeast co‐transformed with BD‐MdPHYB2 and AD‐MdPIF1 grown on SD/−T/−L and SD/−T/−L/−H/−A (+X‐α‐gal) selective medium. (B) MdPHYB2 and MdPIF1 interaction analysis by LCI assay. The image is a representative image of tobacco leaves after infiltration. (C) Quantitative analysis of the relative luminescence intensity in (B). (D) MdPHYB2 and MdPIF1 interaction analysis by BiFC assay. MdPHYB2 was fused to the N‐terminus of yellow fluorescent protein (nYFP), and MdPIF1 was fused to the C‐terminus of YFP (cYFP). The recombinant plasmid was transformed into tobacco leaves. Scale bar = 100 μm. (E) Subcellular localization analysis of the MdPIF1 protein. The *35S::GFP* and *35S::MdPIF1‐GFP* constructs were, respectively, injected into tobacco leaves. Scale bar = 100 μm. (F) The fruit coloration of ‘Huashuo’ apples after ALA treatment. The control was subjected to deionized water. ALA concentration was 200 mg L^−1^. The fruits were continuously illuminated under 200 µmol m^−2^ s^−1^ light at 17°C for 72 h. Scale bar = 4 cm. (G) Relative expression of *MdPIF1* in apple fruit skin in (F). *MdACTIN* was used as an internal reference. (H) Relative expression of *MdPIF1* in apple fruit skin in (F). *MdACTIN* was used as an internal reference. (I) Effect of ALA on *MdPIF1* promoter activity by GUS assay in ‘Orin’ calli. ‘Orin’ calli transformed with the *ProMdPIF1*‐*GUS* were treated with 250 μg L^−1^ ALA or not (Control). Relative *GUS* expression. (J) Effect of ALA on *MdPIF1* promoter activity by LUC assay in tobacco leaves. 0.5 mg L^−1^ ALA was used. Relative LUC fluorescence intensity. All experiments were replicated three times. Values are means ± SEs (*n* = 3). Different letters denote significant differences (one‐way (C), or two‐way (G and H) ANOVA followed by Tukey's test, *p* < 0.05). Asterisks indicate significant differences (I and J; Student's *t*‐test, **p < 0.01). [Color figure can be viewed at wileyonlinelibrary.com]

We then verified the MdPHYB2‐MdPIF1 interaction using LCI and BiFC assays. Strong luciferase activity was observed in tobacco co‐expressing MdPHYB2‐nLUC and cLUC‐MdPIF1 exclusively under light conditions, which showed no fluorescence signal in darkness (Figure 3B,C). In the BiFC assay, no yellow fluorescence was detected when nYFP‐MdPHYB2 and cYFP‐MdPIF1 were co‐expressed in tobacco in darkness. Conversely, robust fluorescence emerged under light conditions (Figure 3D), revealing a light‐dependent MdPHYB2‐MdPIF1 interaction *in planta*.

### 3.6 MdPIF1 Negatively Regulates ALA‐Induced Anthocyanin Accumulation

OE‐*MdPHYB2* depressed *MdPIF1* expression, whereas RNAi‐*MdPHYB2* increased it in transgenic calli (Supporting Information S1: Figure S7). Exogenous ALA inhibited *MdPIF1* expression in all genotypes, with a more pronounced suppression in OE‐*MdPHYB2* lines and an attenuated effect in RNAi‐*MdPHYB2* lines. These results hint that MdPIF1 may be a negative regulator of ALA‐induced anthocyanin accumulation.

To characterize the MdPIF1 function, we performed bioinformatic analysis. A phylogenetic tree constructed based on protein sequences from eight AtPIFs (*A*. *thaliana*), seven MdPIFs (*M*. × *domestica*, apple), five RhPIFs (*Rosa hybrida*, rose), and five VvPIFs (*V. vinifera*, grape) categorized these proteins into four subgroups, with MdPIF1 falling into Group I alongside other PIF1 orthologues (Supporting Information S1: Figure S8A, Supporting Information S2: Table S6). Sequence alignment within Group I identified a highly conserved basic helix‐loop‐helix (bHLH) domain in MdPIF1, as well as active phyB‐binding (APB) and active phyA‐binding (APA) motifs (Supporting Information S1: Figure S8B). Subcellular localization analysis showed that the MdPIF1‐GFP fusion protein was predominantly localized in the nucleus, whereas the control GFP distributed throughout the nucleus, cytoplasm, and cell membrane (Figure 3E), confirming that MdPIF1 is nuclear‐localized.

We next examined *MdPIF1* expression. The results showed that compared with the control, ALA significantly suppressed the *MdPIF1* expression during fruit anthocyanin accumulation (Figure 3F–H). Histochemical staining of *ProMdPIF1‐GUS* in the transgenic apple calli showed that ALA markedly decreased the GUS staining and *GUS* expression (Figure 3I). Consistently, transient expression of *ProMdPIF1‐LUC* in tobacco leaves followed by ALA treatment resulted in a significant decrease in LUC intensity (Figure 3J). These results indicate that ALA inhibits *MdPIF1* promoter activity and gene expression during apple coloration (Figure 3F).

To determine MdPIF1 function, we generated OE‐ and RNAi‐*MdPIF1* constructs. Transient transformation in apple fruits showed that OE*‐MdPIF1* apples decreased coloration with less anthocyanin accumulation, compared with the EV control, whereas RNAi‐*MdPIF1* fruits exhibited a 3.7‐fold increase (Figure 4A–C). ALA treatment significantly increased the anthocyanin content in all genotypes, yet its effect was compromised in OE‐*MdPIF1* but further enhanced in RNAi‐*MdPIF1*. Experiment with transiently transgenic ‘GL‐3’ leaves showed similar results (Figure 4D–F), indicating MdPIF1 negatively regulating ALA‐induced anthocyanin accumulation.

**Figure 4:**
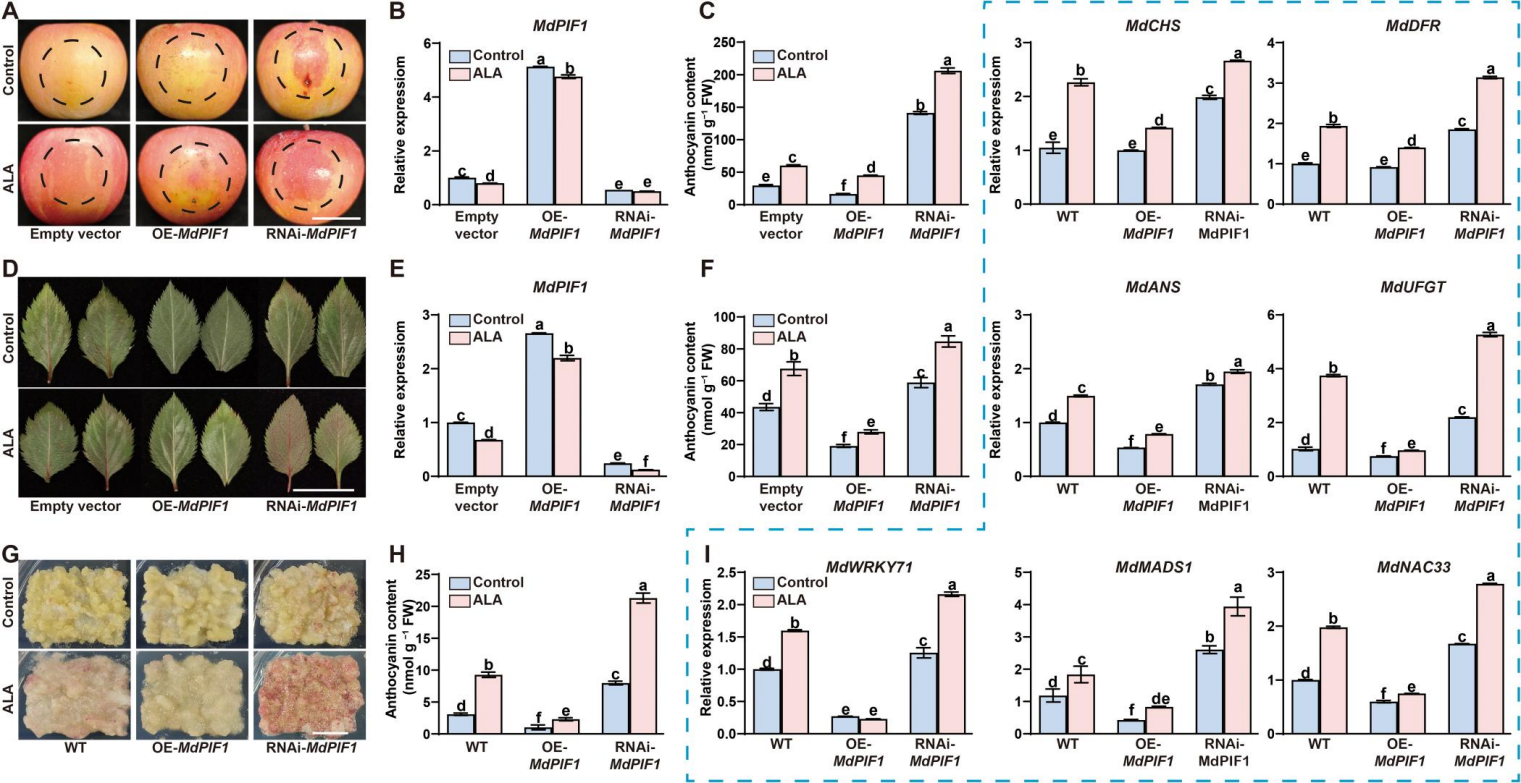
MdPIF1 negatively regulates ALA‐induced anthocyanin accumulation in apple. (A) Transient transformation of apple peel. Debagged ‘Fuji’ apple fruits were injected with a carrier, treated with or without 200 mg L^−1^ ALA and then incubated under constant light (200 μmol m^−2^ s^−1^) at 17°C. OE‐*MdPIF1*: Overexpressing *MdPIF1*. RNAi‐*MdPIF1*: Interfering with the expression of *MdPIF1*. Fruits were light‐exposed for 5 days to ensure coloration. The dashed circles point to the injection sites. Scale bar = 4 cm. (B) *MdPIF1* relative expression in the injection sites of (A). (C) Anthocyanin content in the injection sites of (A). (D) Phenotype of OE‐*MdPIF1* and RNAi‐*MdPIF1* transiently transformed apple leaves treated with or without 25 μg L^−1^ ALA for 3 days. Scale bar = 1 cm. (E) *MdPIF1* relative expression in (D). (F) Anthocyanin content in (D). (G) Phenotype of OE‐*MdPIF1* and RNAi‐*MdPIF1* stable transgenic ‘Orin’ calli treated with or without 250 μg L^−1^ ALA for 2 weeks. Scale bar = 1 cm. (H) Anthocyanin content in (G). (I) The relative expression of *MdWRKY71*, *MdMADS1*, *MdNAC33*, *MdCHS*, *MdDFR*, *MdANS*, and *MdUFGT* in (G). All experiments were replicated three times. Values are means ± SEs (*n* = 3). Different letters denote significant differences (two‐way ANOVA followed by Tukey's test, *p* < 0.05). [Color figure can be viewed at wileyonlinelibrary.com]

Moreover, we generated stable OE‐ and RNAi‐*MdPIF1* ‘Orin’ callus lines, which were identified by PCR and RT‐qPCR (Supporting Information S1: Figure S9). Upon ALA treatment, RNAi‐*MdPIF1* calli displayed enhanced red coloration and higher anthocyanin content, whereas OE‐*MdPIF1* calli remained pale and less responsive to ALA (Figure 4G,H). Expression analysis revealed that the key TF genes (*MdWRKY71*, *MdMADS1*, *MdNAC33*), as well as the structural genes (*MdCHS*, *MdDFR*, *MdANS*, *MdUFGT*) were downregulated in OE‐*MdPIF1* calli but upregulated in RNAi‐*MdPIF1* (Figure 4I). ALA enhanced these gene expressions with its effects compromised in OE‐*MdPIF1* calli. These results confirm that MdPIF1 acts as a negative regulator in ALA‐activated expression of these TF/structural genes during anthocyanin accumulation.

### 3.7 MdPIF1 Directly Binds to E‐Boxes in the Promoters of Key TF Genes

To elucidate how MdPIF1 negatively regulates anthocyanin accumulation, we analyzed the promoter sequences (2000 bp) of the genes related to anthocyanin biosynthesis. Results showed that the promoters of *MdWRKY71*, *MdMADS1*, *MdNAC33, MdPAL*, *MdCHS*, *MdF3H*, *MdANS*, and *MdUFGT* contained at least one E‐box (CANNTG) (Figure 5A, Supporting Information S1: Figure S10A). Y1H assays showed that MdPIF1 bound to the promoters of *MdWRKY71, MdMADS1*, and *MdNAC33* (Figure 5A), but not to all the structural genes (Supporting Information S1: Figure S10B). Using both LUC and GUS reporter systems, we observed that MdPIF1 negatively regulated the promoter activities of these TF genes (Figure 5B–E) but not those of the structural genes (Supporting Information S1: Figure S10C–E). The results of EMSA further demonstrated that MdPIF1 directly and specifically bound to the E‐box elements in promoters of *MdWRKY71* (at the CACATG element, E‐box 1), *MdMADS1* (at the CACATG and CATGTG elements, E‐box 2 and E‐box 3), and *MdNAC33* (at the CACATG element, E‐box 4) (Figure 5F). Collectively, the data from in vitro DNA‐binding assays (EMSA), heterologous reporter systems (Y1H, LUC, GUS), and transgenic analyses indicate that MdPIF1 functions as a transcriptional repressor, directly binding to the promoters of key TF genes (*MdWRKY71*, *MdMADS1* and *MdNAC33*) to inhibit their expression. Yet, it cannot directly bind to the promoters of the structural genes for anthocyanin biosynthesis.

**Figure 5:**
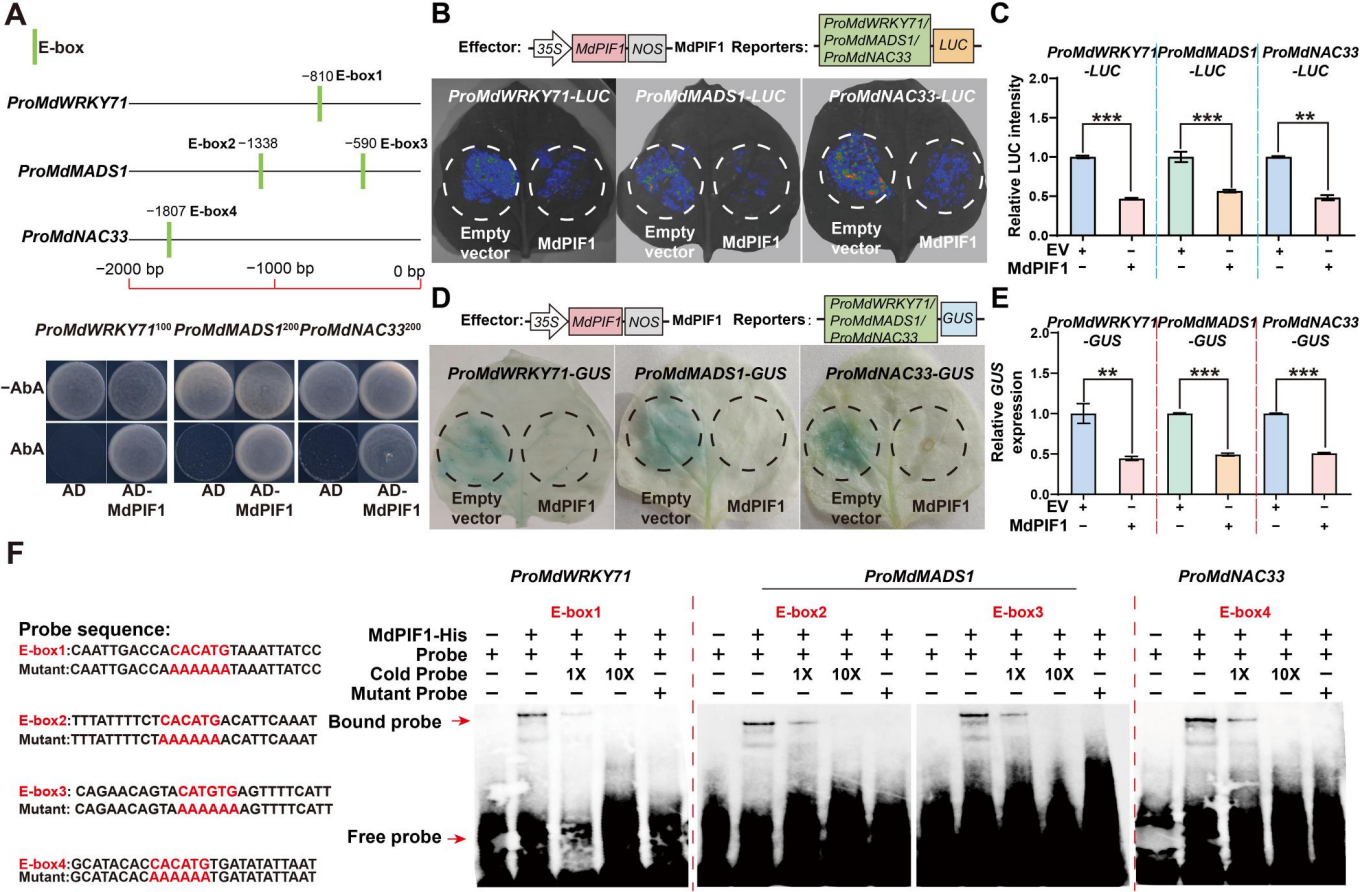
MdPIF1 binds to the promoters of *MdWRKY71*, *MdMADS1* and *MdNAC33* at E‐boxes to negatively regulate their transcriptional activity. (A) Y1H assays show interactions between MdPIF1 and promoters of *MdWRKY71*, *MdMADS1* and *MdNAC33*. A 2000 bp fragment of the *MdWRKY71*, *MdMADS1* and *MdNAC33* promoters was fused to the pAbAi vector as bait. MdPIF1 was fused to the pGADT7 vector as a prey (AD‐MdPIF1). The transformants were grown on selective medium (SD/−Leu) with or without AbA. The superscript numbers in the upper right corner represent AbA concentrations (ng mL^−1^). (B) LUC reporter assay shows the negative effect of MdPIF1 on *MdWRKY71*, *MdMADS1* and *MdNAC33* promoter activity. (C) Relative LUC intensity in (B). (D) GUS reporter assay shows the negative effect of MdPIF1 on *MdWRKY71*, *MdMADS1* and *MdNAC33* promoter activity. (E) Relative *GUS* expression in (D). (F) EMSA shows that MdPIF1 binds directly to the E‐box on the *MdWRKY71*, *MdMADS1* and *MdNAC33* promoters. All experiments were replicated three times. Values are means ± SEs (*n* = 3). Asterisks indicate significant differences (Student's *t*‐test, **p < 0.01, ***p < 0.001). [Color figure can be viewed at wileyonlinelibrary.com]

### 3.8 MdPHYB2 Interferes With MdPIF1‐DNA Binding to Derepress Gene Expression

To elucidate how MdPHYB2 antagonizes MdPIF1 in regulating ALA‐induced apple anthocyanin biosynthesis, we performed LUC reporter assays in tobacco leaves under both dark and light conditions. Compared to the control, MdPIF1 always inhibited the transcriptional activity of the *MdWRKY71*, *MdMADS1*, and *MdNAC33* promoters. Under dark conditions, MdPHYB2 did not affect the depressive effects of MdPIF1 on the promoters. Under light condition, the inhibition of MdPIF1 on the promoters was significantly alleviated by co‐expression of *35S::MdPHYB2* (Figure 6A–C). These results indicate that MdPHYB2 alleviates the inhibitory effect of MdPIF1 on downstream TF genes, which is light‐dependent.

**Figure 6:**
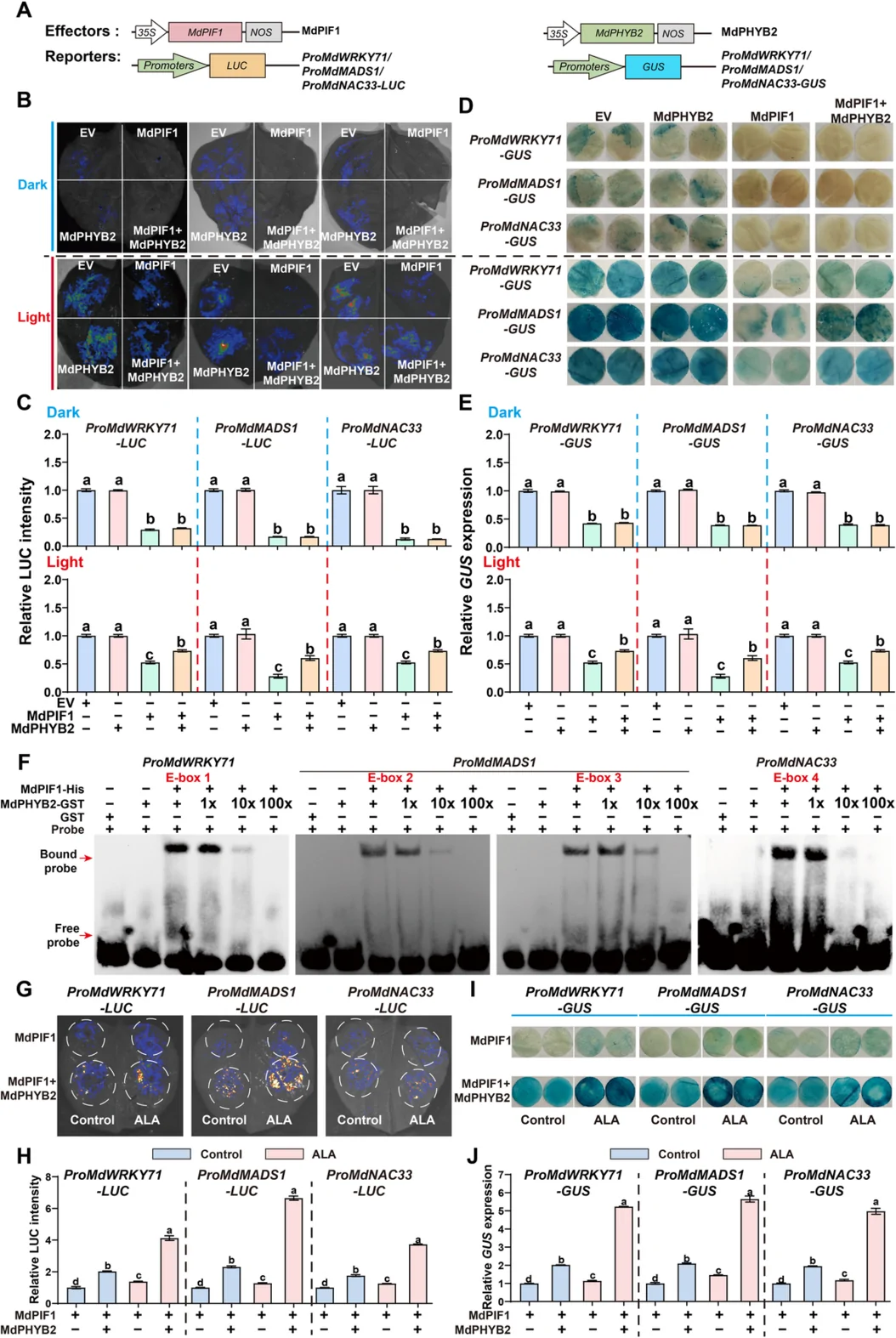
MdPHYB2 suppresses the transcriptional inhibitory effect of MdPIF1 on *MdWRKY71*, *MdMADS1* and *MdNAC33*. (A) Schematic diagram of the effector vectors (*35S::MdPIF1* and *35S::MdPHYB2*) and LUC/GUS reporter vectors (*ProMdWRKY71‐LUC/GUS, ProMdMADS1‐LUC/GUS*, *and ProMdNAC33‐LUC/GUS*). (B) Effect of MdPHYB2 on transcriptional regulatory activity of MdPIF1 by LUC assay. (C) Relative LUC fluorescence intensity in (B). (D) Effect of MdPHYB2 on transcriptional regulatory activity of MdPIF1 by GUS assay. (E) Relative *GUS* expression in (D). (F) EMSA experiments verify the effect of MdPHYB2 on the binding strength of MdPIF1 to the *MdWRKY71, MdMADS1* and *MdNAC33* promoters. (G) Effects of MdPHYB2 and MdPIF1 on downstream target gene promoter activity after 0.5 mg L^−1^ ALA treatment by LUC assay. (H) Relative LUC intensity in (G). (I) Effects of MdPHYB2 and MdPIF1 on the promoter activity of downstream target genes after 0.5 mg L^−1^ ALA treatment by GUS assay. (J) Relative *GUS* expression in (I). All experiments were replicated three times. Values are means ± SEs (*n* = 3). Different letters in each panel denote significant differences (one‐way ANOVA followed by Tukey's test, *p* < 0.05). [Color figure can be viewed at wileyonlinelibrary.com]

Further GUS reporter assays corroborated these findings. In darkness, MdPIF1 repressed the target promoter activity without interference from MdPHYB2. In contrast, under light, MdPHYB2 largely eliminated the repressive activity of MdPIF1‐targeted promoters (Figure 6D,E).

To delve deeper into the mechanism by which MdPHYB2 modulates the activity of MdPIF1, we performed EMSA using recombinant MdPHYB2‐GST and MdPIF1‐HIS proteins and DNA probes containing E‐box elements from *MdWRKY71*, *MdMADS1* and *MdNAC33* promoters. MdPIF1‐HIS bound to the E‐box‐containing probe, but MdPHYB2‐GST gradually reduced MdPIF1 binding as the concentrations increased (Figure 6F). These results demonstrate that MdPHYB2 antagonizes MdPIF1 by interfering with its DNA‐binding activity, thereby derepressing downstream target TF gene expression.

To verify this antagonistic relationship *in planta*, we performed transient co‐overexpression assays in both apple fruits and leaves. We co‐infiltrated *Agrobacterium* strains carrying four different constructs: EV, OE‐*MdPIF1*, OE‐*MdPHYB2*, and OE‐*MdPIF1* + OE‐*MdPHYB2*. In both tissues, OE‐*MdPIF1* significantly suppressed anthocyanin accumulation while OE‐*MdPHYB2* significantly increased the pigment content, compared to the EV control (Supporting Information S1: Figure S11). Co‐expression of *MdPHYB2* with *MdPIF1* significantly restored anthocyanin levels, attenuating the suppressive effect of *MdPIF1*. These results provide direct functional evidence that MdPHYB2 counteracts MdPIF1‐mediated repression of anthocyanin biosynthesis in apple fruits and leaves.

Furthermore, in ‘Orin’ calli, we observed that ALA had minimal effect on the expression of *MdPHYB2* and *MdPIF1* under dark conditions (Supporting Information S1: Figure S12). However, ALA rapidly upregulated *MdPHYB2* and downregulated *MdPIF1* within 1 h, with the effects persisting for at least 12 h, under light conditions. Therefore, ALA‐induced upregulation of *MdPHYB2* and downregulation of *MdPIF1* occur only in the light, indicating that light provides the necessary permissive context for these transcriptional changes to drive anthocyanin biosynthesis.

In addition, LUC/GUS reporter assays confirmed that *MdPHYB2* can suppress the inhibition of *MdPIF1* on the promoters of the target genes, including *MdWRKY71*, *MdMADS1* and *MdNAC33*, when *MdPHYB2* was co‐expressed with *MdPIF1*. When ALA was added, the promotive effects were more pronounced (Figure 6G–J). These results indicate that ALA potentiates MdPHYB2‐attenuating MdPIF1 repression on the downstream gene expression.

## 4 Discussion

### 4.1 Functional Characterization of Phytochrome Genes in Apple

As photoreceptors, PHYs perceive red and far‐red light to transmit signals that regulate diverse plant growth and developmental processes (Cheng et al. 2021), including stomatal movement (Wang et al. 2010), anthocyanin biosynthesis (Li et al. 2014; Gao et al. 2023), thermomorphogenesis (Jung et al. 2016), hormone signalling (Zhao et al. 2022), salt tolerance (Han et al. 2023) and photomorphogenesis (Mu et al. 2024). *Arabidopsis* possesses five members of PHY (PHYA‐PHYE) (Li et al. 2015), while rice has three (PHYA‐PHYC) (Sun et al. 2017). In maize (*Z. mays*), six *PHY* genes fall into three subgroups: *ZmPHYA1/A2*, *ZmPHYB1/2* and *ZmPHYC1/C2* (Cao et al. 2024). Here, in apple, seven *MdPHY* genes can be clustered into four subgroups: *MdPHYA*, *MdPHYB*, *MdPHYC* and *MdPHYE*, lacking a distinct *MdPHYD* (Supporting Information S1: Figure S1). In *Arabidopsis*, PHYD and PHYE share more than 80% amino acid identity with PHYB (Clack et al. 1994). PHYB functions as the major phytochrome under continuous red light, while PHYD appears non‐essential in some environments (Aukerman et al. 1997). Consistent with this, PHYD is absent from apple, peach, grape, tomato, tea, and tobacco genomes, indicating that PHYD loss is common among dicots. Therefore, the *PHY* gene family follows a conserved evolutionary trajectory, with lineage‐specific variations.

The MdPHY proteins comprise three conserved photosensory domains (PAS_2, GAF and PHY) at the N‐terminus and dimerization domains (two PAS repeats, histidine kinase and HATPase_c) at the C‐terminus (Supporting Information S1: Figure S1C). This structure is highly conserved among MdPHYs and matches the established *Arabidopsis* model (Cheng et al. 2021). In *Arabidopsis*, the N‐terminal PHY domain binds phytochromobilin to sense light and drive Pr/Pfr interconversion, whereas the C‐terminal output module mediates dimerization and signal transmission to downstream effectors. Reversible isomerization at the C15─C16 bond of phytochromobilin generates the inactive Pr in darkness and the active Pfr under light (Bae and Choi 2008). Conformational switching between Pr and Pfr transmits light signals to downstream responses, a mechanism that is widely conserved across plant species.

Phytochromes function in the nucleus as homo‐ or heterodimers (Matsushita et al. 2003). We observed that MdPHYA1 and MdPHYC form only homodimers, while other MdPHYs form both homo‐ and heterodimers (Figure 1B). Notably, MdPHYB2 interacts with MdPHYA2, MdPHYB1 and MdPHYE1, and MdPHYE1 similarly forms heterodimers with MdPHYB1, MdPHYB2, and MdPHYE2. This complex interaction network likely underpins the functional plasticity of the apple PHY system and provides a framework for future mechanistic studies. It should be pointed out that MdPHY dimer formation is light‐independent, because we found that MdPHYB2 can self‐interact under both dark and light conditions (Figure 1B; Supporting Information S1: Figure S2A). Furthermore, in the BiFC assay with tobacco leaves under light conditions, we observed that the yellow fluorescence formed by self‐interaction of MdPHYB2 is a circular spot, highly resembling within the cell nucleus, implying MdPHYB2 homodimer is localized in the nucleus upon light exposure (Supporting Information S1: Figure S2A). Subcellular localization revealed that all MdPHYs are scattered in the cytosol in darkness but translocated into the nucleus under light (Figure 1C). Cytoplasmic PHYs are in the inactive Pr form, whereas light converts them into the active Pfr form (Chen et al. 2005). This light‐dependent conformational switch and nuclear translocation, which facilitates the interaction of PHYs with PIFs to transduce light signals, have been characterized in *Arabidopsis* (Rockwell et al. 2006; Cheng et al. 2021). In the present study, we analyzed the responses of the *MdPHY* promoters to different stimuli, including ALA, ABA, NaCl and MeJA. The results showed the promoters of all *MdPHYs* except *MdPHYA2* are responsive to at least one stimulus, among which *MdPHYB2* promoter activity is enhanced by ALA and ABA but inhibited by NaCl and MeJA (Supporting Information S1: Figure S13), indicating that *MdPHY* expression is modulated by multiple environmental cues.

### 4.2 MdPHYB2 Functions as a Key Regulator in ALA‐Induced Anthocyanin Accumulation

PHYs are known to regulate anthocyanin biosynthesis across species, such as tomato (Husaineid et al. 2007), *Arabidopsis* (Li et al. 2014), and woodland strawberry (Gao et al. 2023). ALA, an essential precursor of all tetrapyrrole compounds, including phytochromobilin (Wang et al. 2023; Wang, Zhang, Zhao, et al. 2025), has garnered considerable attention for its capacity to enhance anthocyanin accumulation (Zhang, Zhang, et al. 2024; Zhang et al. 2025). It is known that both ALA biosynthesis and catabolism are light‐dependent (von Wettstein et al. 1995); however, whether a process regulated by ALA is light‐dependent is seldom reported. Given that phytochromobilin is derived from ALA and the functions of PHYs are dependent on phytochromobilin (Montgomery et al. 2001), Wang et al. (2004) speculated that MdPHYs might be involved in ALA‐promoted coloration in apple. It should be noted, however, that whether ALA influences PHYB activity through chromophore biosynthesis remains speculative, as direct evidence for changes in phytochromobilin accumulation, PHYB photostate, PHYB nuclear localization, or MdPHYB2–MdPIF1 interaction is lacking.

In the present study, we found that among the seven *MdPHYs*, only *MdPHYB2* expression was consistently upregulated during ALA‐induced anthocyanin accumulation. OE‐*MdPHYB2* markedly enhanced ALA's stimulatory effect, whereas RNAi*‐MdPHYB2* completely eliminated the effect (Figure 2). This underscores the dominant role of MdPHYB2 in the ALA‐induced anthocyanin biosynthesis. Yet, the potential roles of other MdPHY members cannot be excluded. Because PHYA is reported to mediate far‐red light responses and influence pigment deposition in canopy shade (Kunkel et al. 1996). PHYC is linked to photoperiodic regulation (Woods et al. 2023). Different PHY members may act in a light‐quality‐, developmental stage‐, or tissue‐specific manner to finely tune anthocyanin biosynthesis. The complex and potentially distinct regulation of other MdPHY members needs further study.

Furthermore, how ALA upregulates *MdPHYB2* expression remains unclear. They are the events upstream of *MdPHYB2* expression, very important but seldom reported. Recently, Yin et al. (2025) proposed that ALA promotes *MdFC2* expression, which is responsible for heme biosynthesis and affects ALA‐induced anthocyanin accumulation in apples. This indicates that ALA's regulatory role in the heme biosynthesis pathway is crucial for phytochrome biosynthesis and anthocyanin accumulation, which is under investigation.

### 4.3 MdPHYB2‐MdPIF1 Module Regulates ALA‐Induced Apple Anthocyanin Accumulation

In the nucleus, PHYs transduce light signals by interacting with various proteins, including PIFs (Ma et al. 2023). In this study, we employed Y2H library screening with MdPHYB2 as bait and identified MdPIF1 as a direct interacting partner from 63 potential proteins (Supporting Information S2: Table S5), which was confirmed by Y2H, LCI, and BiFC (Figure 3). Bioinformatic analysis revealed that MdPIF1 belongs to subgroup I among the four MdPIF subgroups in the apple genome, and its protein sequence contains typical APB, APA, and bHLH domains. Furthermore, subcellular localization assays demonstrate that MdPIF1 is localized in the nucleus, which aligns with its predicted function as a TF. Notably, during ALA‐induced apple fruit coloration, *MdPIF1* expression was down‐regulated compared with the control. Consistent with this, GUS and LUC assays confirmed a reduction in its promoter activity by ALA treatment, indicating that ALA inhibits *MdPIF1* expression by suppressing its promoter activity. Functionally, in detached apple fruits, leaves, and callus cells, OE‐*MdPIF1* inhibited while RNAi‐*MdPIF1* promoted anthocyanin accumulation (Figure 4). Furthermore, exogenous ALA promoted anthocyanin accumulation very strongly in the RNAi‐*MdPIF1* but minimally in the OE‐*MdPIF1*. These results collectively demonstrate that MdPIF1 acts as a negative TF in ALA‐induced anthocyanin accumulation, and this repression is alleviated by ALA‐induced upregulation of MdPHYB2. Under light, ALA upregulates *MdPHYB2* expression while downregulating *MdPIF1* (Supporting Information S1: Figure S12). This reciprocal regulatory expression pattern occurs only under light conditions, which contributes to the acceleration of ALA‐promoted anthocyanin accumulation. The detailed mechanisms underlying the light‐dependent transcriptional regulation warrant further investigation. In addition, the altered *MdPIF1* expression observed in the *MdPHYB2* transgenic calli (Supporting Information S1: Figure S7) suggests that, beyond the direct protein–protein interaction with MdPIF1, MdPHYB2 may affect *MdPIF1* transcription through some unknown mechanisms, which need to be elucidated further.

Previous studies have confirmed that *MdWRKY71*, *MdMADS1*, and *MdNAC33* respond to ALA signals through the non‐MBW pathway and the MBW pathway, respectively, positively regulating apple anthocyanin biosynthesis (Zhang, Zhang, et al. 2024; Zhang et al. 2025). Additionally, the structural genes *MdPAL*, *MdCHS*, *MdF3H*, *MdDFR*, *MdANS*, and *MdUFGT*, as well as the transport gene *MdGSTF12*, are also upregulated by ALA (Fang et al. 2022, 2023). Similar responses in different transgenic apple calli were observed in the current study, where OE‐*MdPIF1* depressed while RNAi‐*MdPIF1* promoted the gene expression with anthocyanin accumulation (Figure 4I). These findings indicate that MdPIF1 functions as a transcriptional repressor of genes related to anthocyanin accumulation. Protein‐nucleotide interacting experiments confirm that MdPIF1 can directly bind to the E‐box elements in the promoters of *MdWRKY71*, *MdMADS1*, and *MdNAC33*, inhibiting their expression, but not to the promoters of structural genes (Figure 5, Supporting Information S1: Figure S10). These indicate that MdPIF1 suppresses anthocyanin biosynthesis by transcriptionally repressing the target TF genes, which then coordinately downregulate the downstream structural gene expression and anthocyanin accumulation. Interestingly, ALA significantly induces the downstream gene expression in the RNAi*‐MdPIF1* lines (Figure 4I), suggesting the existence of either MdPIF1‐independent pathways or functionally redundant repressors that are simultaneously suppressed by ALA. In *Arabidopsis*, PIF4 directly binds to the promoter of MYB TF *PAP1* and represses its transcription, thus inhibiting anthocyanin synthesis (Liu, Wang, et al. 2021). Xie et al. (2013) reported that ALA promoted *MdMYBa/10*, *MdbHLH3*, and *MdWD40* expression. Zhang, Zhang, et al. (2024) pointed out that ALA‐promoted *MdbHLH33* expression was indirectly upregulated by MdNAC33. Additionally, MdNAC33 can interact with MdMYB1, stimulating the latter directly to transcriptionally upregulate *MdUFGT* and *MdGSTF12*, facilitating anthocyanin accumulation. Collectively, our findings support a model in which MdPIF1 acts as the primary repressor of the downstream TF genes, such as *MdWRKY71*, *MdMADS1* and *MdNAC33* on the one hand, and on the other hand, is antagonized by MdPHYB2 under light conditions, coordinately mediating ALA‐induced apple anthocyanin accumulation.

A recent study reported that another *MdPIF1* allele (MD10G1170600) enhanced anthocyanin biosynthesis (Zhang, Li, et al. 2024), which is different from our findings with KU255859.1/MDP0000289642 (Figure 4). Sequence comparison revealed that our MdPIF1 contains an additional 106 amino acids at the C‐terminus (Supporting Information S1: Figure S14), which likely accounts for this functional divergence. This structural difference may alter protein conformation and DNA‐binding specificity, converting MdPIF1 from an activator to a repressor. Future studies should confirm whether this C‐terminal extension is responsible for the opposite phenotypes.

Several studies have found that light‐induced nuclear translocation and binding of PHYs to PIFs trigger the phosphorylation and rapid ubiquitin‐dependent degradation of the latter (Paik et al. 2019; Song et al. 2023). Beyond this, the active Pfr form of PHYs acts upstream of PIFs by inhibiting their DNA‐binding activity (Park et al. 2012). Consistent with this model, we observed that the co‐transformation of *MdPHYB2* and *MdPIF1* alleviates the inhibitory effect of MdPIF1 on the promoter activity of target genes, including *MdWRKY71*, *MdMADS1*, and *MdNAC33* (Figure 6). Furthermore, EMSA results revealed that MdPHYB2 can competitively antagonize MdPIF1 binding to the E‐box elements in the promoters of these genes in vitro. These results provide initial evidence that MdPHYB2 interferes with MdPIF1 binding to the promoters of downstream anthocyanin biosynthetic genes, although the underlying mechanism requires further elucidation. Beyond MdPIF1, we found that MdPHYB2 also interacted with the other six MdPIF members (Supporting Information S1: Figure S15), suggesting broader regulatory roles that warrant further investigation. In addition, our preliminary results showed that neither MdPHYB2 nor MdPIF1 directly interacts with MdMYB1, MdbHLH3, or MdHY5, and that MdPIF1 represses *MdMYB1* and *MdbHLH3* promoter activities but not that of *MdHY5* (Supporting Information S1: Figure S16). These initial observations await further validation.

## 5 Conclusion

Our previous works identified MdNAC33 (Zhang, Zhang, et al. 2024) and the MdWRKY71‐MdMADS1 module (Zhang et al. 2025) as key mediators of ALA‐induced anthocyanin accumulation through the MBW‐dependent pathway and MBW‐independent pathways, respectively. The present study reveals that MdPHYB2 acts as a critical ALA‐responsive regulator that suppresses MdPIF1 activity, thereby transducing the light signal to the nucleus, releasing the repression on anthocyanin biosynthetic genes. Integrating these findings, we propose a unified model depicting how ALA acts in a light‐dependent manner on the MdPHYB2‐MdPIF1 molecular module, which in turn regulates anthocyanin accumulation in apple (Figure 7). This model provides a visual representation of the intricate molecular interactions involved. Our findings not only advance our understanding of how ALA acts in a light‐dependent manner to regulate fruit pigmentation but also identify a precise genetic target for apple quality improvement. The MdPHYB2‐MdPIF1 module offers biotechnological potential to optimize anthocyanin profiles under specific light regimes, enabling the development of apple varieties with enhanced nutritional and market value.

**Figure 7:**
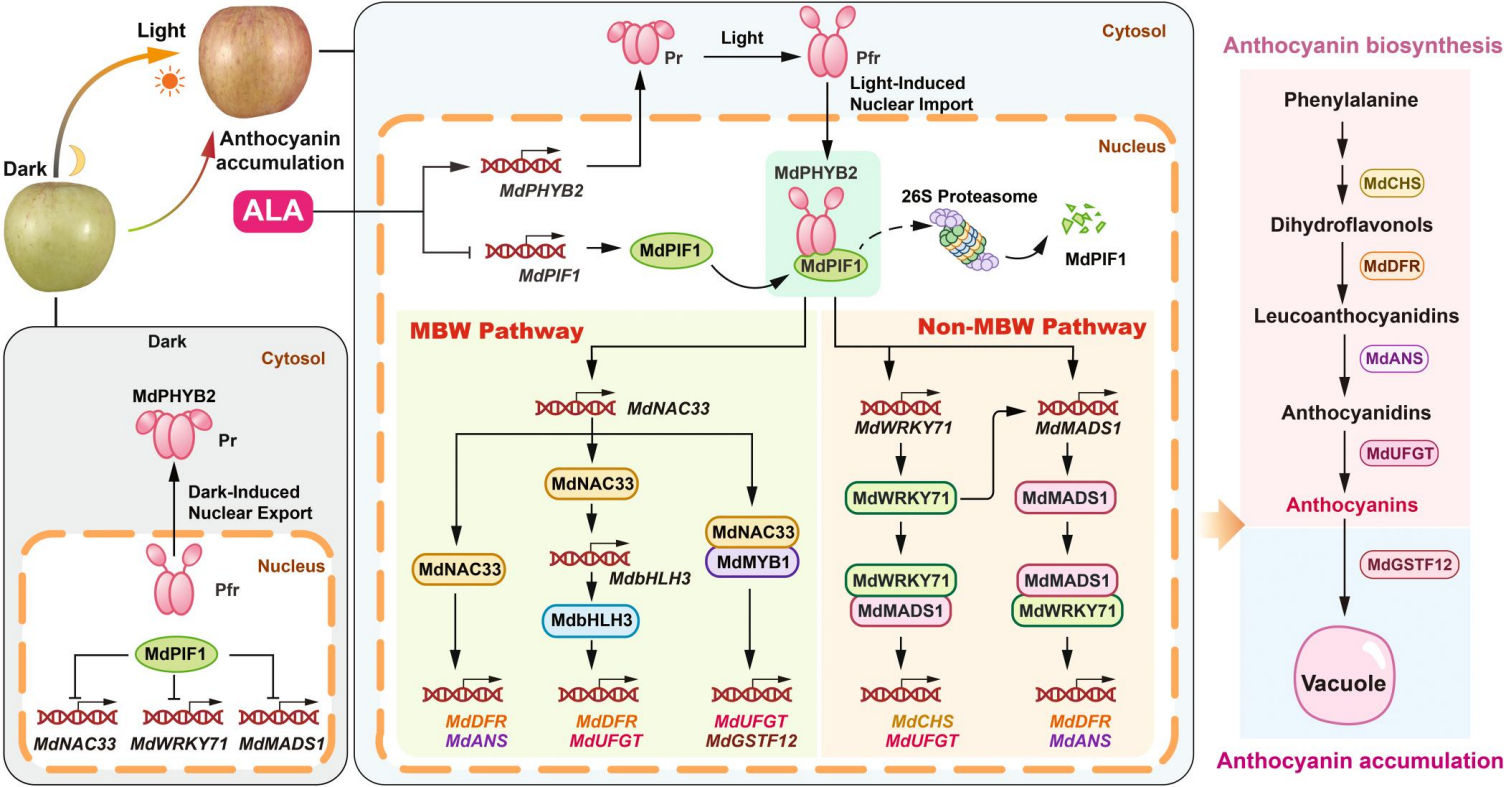
A working model of the MdPHYB2‐MdPIF1 module in anthocyanin accumulation induced by ALA in a light‐dependent manner. Under dark conditions, MdPHYB2 is localized in the cytosol and inactive with the C15‐*Z*, *anti* conformation (Pr), while the highly expressed TF MdPIF1 in the nucleus represses expression of *MdNAC33*, *MdWRKY71* and *MdMADS1*, thereby anthocyanin biosynthesis is blocked. Upon light exposure, exogenous ALA upregulates *MdPHYB2* expression and downregulates *MdPIF1* expression. The enhanced MdPHYB2 protein light‐dependently converses into the C15‐*E*, *anti* conformation (Pfr) and shifts from the cytosol to the nucleus, where it interacts with the ALA‐downregulated expressed MdPIF1. The interaction of MdPHYB2 with MdPIF1 sensitizes the latter to be degraded, releasing the transcriptional inhibition on downstream genes. Consequently, the expression of TF genes such as *MdNAC33*, *MdWRKY71* and *MdMADS1* is activated. Then, the upregulated expressed MdNAC33 can further upregulate downstream gene expression, including TF genes (*MdbHLH3* and *MdMYB1*), structural genes (*MdDFR*, *MdANS*, *MdUFGT*) and transporter gene (*MdGSTF12*), facilitating anthocyanin accumulation through an MBW pathway (Zhang, Zhang, et al. 2024). On the other hand, the upregulated MdWRKY71 can transcriptionally regulate *MdMADS1* expression, then MdWRKY71 and MdMADS1 interact with each other, upregulating expression of *MdCHS* and *MdUFGT*, as well as *MdDFR* and *MdANS* in different modes to promote anthocyanin accumulation. Since the route is independent of the MBW complex, it is termed the non‐MBW pathway (Zhang et al. 2025). The biosynthesized anthocyanins are transported into vacuoles and stored there for fruit coloration. [Color figure can be viewed at wileyonlinelibrary.com]

## Conflicts of Interest

The authors declare no conflicts of interest.

## Supporting information

Supporting File 1:

Supporting File 2:

## Data Availability

The data that support the findings of this study are available on request from the corresponding author.
