# Supplementary material for: MdPHYB2‐MdPIF1 Module Regulates Transcription Factors to Mediate Light‐Dependent ALA‐Induced Apple Anthocyanin Biosynthesis
Source: Plant Cell Environ. 2026 Jul 1;49(11):7808–24. doi: 10.1111/pce.70702 (PMC13646689; doi:10.1111/pce.70702)
Supplement: Supplementary file 1 [file PCE-49-7808-s001.docx]

**Supporting Information：Figure S1—16**

**Figure S1. Bioinformatic analysis of apple phytochrome genes (*MdPHYs*)**. (**A**) Chromosomal locations of 7 *MdPHY* genes. (**B**) Phylogenetic relationships among PHY proteins of different plant species. MEGA-X was used to construct the phylogenetic tree (NJ method, 1000 bootstrap). Md: apple (*Malus × domestica*), At: Arabidopsis (*Arabidopsis thaliana*), Sl: tomato (*Solanum lycopersicum*), Vv: grape (*Vitis vinifera*), Os: rice (*Oryza sativa*), Cs: tea (*Camellia sinensis*), Nt: tobacco (*Nicotiana tabacum*), Zm: maize (*Zea mays*), Pp: peach (*Prunus persica*). Distinct color backgrounds denote the characteristics of the subgroups, with bold font highlighting the positions of the apple PHY members. (**C**) Phylogenetic relationships, domain conservation, and gene structure of *MdPHYs*. From left to right, the diagram shows: the evolutionary tree, the conserved domain predictions, and the exon-intron structure. Numbers above the conserved domains represent the amino acid residue positions relative to the start of each protein. (**D**) Collinearity analysis of the *MdPHY* gene family. The black lines represent homologous gene pairs. (**E**) The distribution pattern of 14 *cis*-acting elements across the promoter regions of *MdPHY* family, with the phylogenetic tree corresponding to that in **C**. (**F**) Quantification of *cis*-acting elements in **E**. Numbers in the box represent the number of different *cis-*acting elements.

**Figure S2. The self-interaction of MdPHYB2 protein under light detected by Y2H, LCI and BiFC.** (**A**) Y2H interaction analysis. Full-length *MdPHYB2* sequences were constructed into BD and AD vectors and co-transformed into Y2Hgold yeast. Interactions were assessed on selective media for 3 days on the light conditions. (**B**) Luc complementation imaging (LCI) assay. Full-length *MdPHYB2* sequences were constructed into nLUC and cLUC vectors and co-transformed into tobacco leaves. (**C**) Bimolecular fluorescence complementation (BiFC) assay. nYFP-*MdPHYB2* and cYFP-*MdPHYB2* were transiently co-expressed in tobacco leaves and their BiFC signals observed under a fluorescence microscope. Scale bar=100 μm.

**Figure S3. ALA fails to promote apple coloration in the dark.** (**A**) Bagged ‘Huashuo’ apples, treated with or without 200 mg L−1 ALA, were continuously kept in the dark at 17°C and photographed at 24, 48, and 72 h. (**B**) Anthocyanin content in apple peel of **A**. Values are means ± standard errors (SEs, n = 3). The different lowercases in a panel represent significant differences (two-way ANOVA followed by Tukey's test, *P* < 0.05). Scale bar = 4 cm.

**Figure S4. The relative expression of *MdPHYs* during ALA-inducing apple coloration under light.** Values are means ± standard errors (SEs, n = 3). The different lowercases in a panel represent significant differences (two-way ANOVA followed by Tukey’s test, *P* < 0.05).

**Figure S5. Identification of the *MdPHYB2*-transformed ‘Orin’ apple calli by western blot, PCR and RT-qPCR.**

**Figure S6. Determination of the relative expression level of *MdPHYB1* in RNAi-*MdPHYB2* transgenic ‘Orin’ apple calli.**

**Figure S7. Determination of the relative expression level of *MdPIF1* in *MdPHYB2* transgenic ‘Orin’ apple call treated with ALA.**

**Figure S8. Bioinformatics analysis of MdPIF1. (A) Phylogenetic tree of PIFs in apple and other species. At: Arabidopsis thaliana, Rh: *Rosa hybrida*, Md: *Malus × domestica*, Vv: *Vitis vinifera*. (B) Sequence alignment analysis between MdPIF1 and PIFs of the same subfamily. APB, the active phyB-binding motif; APA, the active phyA-binding motif; bHLH, basic helix-loop-helix domain.**

**Figure S9. Identification of the *MdPIF1*-transformed ‘Orin’ apple calli by PCR and RT-qPCR.**

**Figure S10. Analysis of E-boxes in the promoters of anthocyanin biosynthesis structural genes *MdPAL*, *MdCHS*, *MdF3H*, *MdANS*, and *MdUFGT*, and interaction analysis of these gene promoters with MdPIF1 by Y1H and LUC assays.**

**Figure S11. Transient co-expression of *MdPHYB2* and *MdPIF1* in apple fruits and leaves.**

**Figure S12. Effects of ALA on *MdPHYs* and *MdPIF1* expression in the ‘Orin’ apple calli cultured under dark or light conditions**

**.**

**Figure S13. Responses of *MdPHY* promoters to different chemicals including ALA, ABA, NaCl and MeJA detected by GUS staining and *GUS* expression.**

**Figure S14. Amino acid sequence alignment between our MdPIF1 (PIF_MDP0000289642) and the previous MdPIF1 (PIF_MD10G1170600).**

**Figure S15. MdPHYB2 interacts with all MdPIF proteins.**

**Figure S16. Preliminary analysis of the relationship between MdPHYB2/MdPIF1 and known anthocyanin regulators (MdMYB1, MdbHLH3, MdHY5).**

(**A-B**) Yeast two-hybrid assays showed no direct interaction between MdPIF1 (or MdPHYB2) and MdMYB1, MdbHLH3, or MdHY5.

(**C**) Promoter *cis*-element analysis showed that E-box motifs are present in the promoters of *MdMYB1* and *MdbHLH3*, but are absent from the *MdHY5* promoter.

(**D-F**) LUC assays showed that MdPIF1 inhibits the activities of the *MdMYB1* and *MdbHLH3* promoters, whereas it does not alter the activity of the *MdHY5* promoter.
